# The Allure of Big Data to Improve Stroke Outcomes: Review of Current Literature

**DOI:** 10.1007/s11910-022-01180-z

**Published:** 2022-03-11

**Authors:** Muideen T. Olaiya, Nita Sodhi-Berry, Lachlan L. Dalli, Kiran Bam, Amanda G. Thrift, Judith M. Katzenellenbogen, Lee Nedkoff, Joosup Kim, Monique F. Kilkenny

**Affiliations:** 1 Stroke and Ageing Research, Department of Medicine, School of Clinical Sciences at Monash Health, Monash University, Clayton, VIC Australia; 2 Cardiovascular Research Group, School of Population and Global Health, The University of Western Australia, Perth, WA Australia; 3 Telethon Kids Institute, The University of Western Australia, Perth, WA Australia; 4 The Florey Institute of Neuroscience and Mental Health, The University of Melbourne, Heidelberg, VIC Australia

**Keywords:** Big data, Stroke, Outcomes, Mortality, Validation studies, Machine learning

## Abstract

**Purpose of Review:**

To critically appraise literature on recent advances and methods using “big data” to evaluate stroke outcomes and associated factors.

**Recent Findings:**

Recent big data studies provided new evidence on the incidence of stroke outcomes, and important emerging predictors of these outcomes. Main highlights included the identification of COVID-19 infection and exposure to a low-dose particulate matter as emerging predictors of mortality post-stroke. Demographic (age, sex) and geographical (rural vs. urban) disparities in outcomes were also identified. There was a surge in methodological (e.g., machine learning and validation) studies aimed at maximizing the efficiency of big data for improving the prediction of stroke outcomes. However, considerable delays remain between data generation and publication.

**Summary:**

Big data are driving rapid innovations in research of stroke outcomes, generating novel evidence for bridging practice gaps. Opportunity exists to harness big data to drive real-time improvements in stroke outcomes.

**Supplementary Information:**

The online version contains supplementary material available at 10.1007/s11910-022-01180-z.

## Introduction

Globally, stroke is a major cause of death and disability [1]. Survivors often report poor quality of life and increased levels of disability, and are at an elevated risk of recurrent vascular events [1, 2]. In recent decades, there has been substantial interest in using large, routinely collected data from various sources, often called “big data,” to generate real-world evidence for understanding and improving outcomes after a stroke or vascular event [3, 4]. Big data–enabled research can be distinguished from other types of research based on the attributes described in the *5Vs* framework, including: *volume* (the size or number of records), *variety* (heterogeneity or diversity of type, structure and setting of data), *velocity* (rapid generation and reporting of data), *veracity* (data quality and reliability), and *variability* (variations between different data sources and datasets) [4, 5]. In stroke research, big data are valuable, low-cost resources for making evidence-based decisions to guide practice and policy on the prevention of adverse outcomes following stroke [4].

Given the rapidly expanding use of big data to explore outcomes following stroke, an updated review of the methods and major findings from these studies is needed to help guide the future directions for big data–enabled research. In this narrative review, we critically appraised literature on recent advances and methods using big data to evaluate health and economic outcomes after stroke and determinants of these outcomes.

## Search Strategy

This was a narrative review of cohort studies on outcomes following discharge for a stroke or transient ischemic attack (TIA). Given the ongoing pandemic, we also reviewed studies of inpatient outcomes in those with concurrent COVID-19 infection. Owing to the breadth of possible outcomes to explore, we focused only on major stroke outcomes, i.e., mortality, hospital readmissions, medication use/adherence, functional measures, quality of life, and healthcare costs. We searched Ovid MEDLINE, Ovid Embase, and CINAHL Plus databases for studies published between January 2019 and August 2021, with outcome data on at least 1,000 people with stroke/TIA, aged ≥ 18 years. Grey literature, government documents, conference abstracts, and articles published in languages other than English were excluded. We further excluded articles which did not meet the attributes of big data research described in the *5Vs* framework [5], or articles considered to be of low quality (score < 6) based on the Newcastle–Ottawa quality assessment scale [6, 7].

## Characterization of Studies on Stroke Outcomes Based on the *5Vs* Framework

**Fig. 1:**
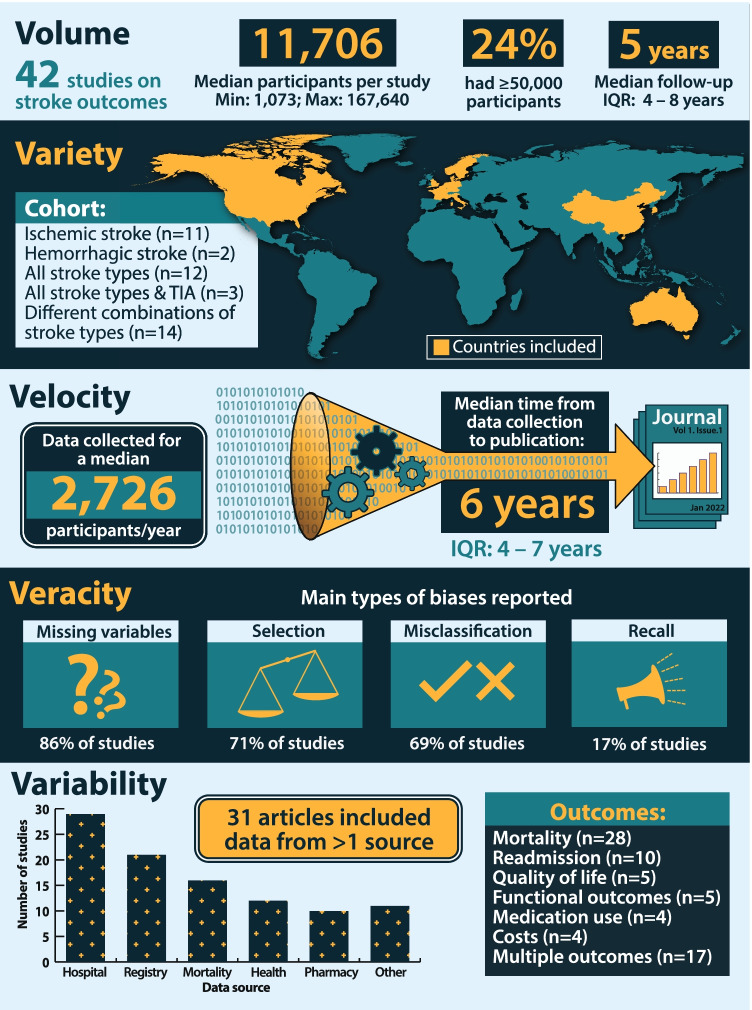
Characterization of studies on stroke outcomes based on the *5Vs* framework

Overall, 58 studies met the inclusion criteria, including 42 studies (72%) of stroke outcomes (Supplementary Table I). There were an additional 16 studies (28%) focused on analytic methods or validation studies used to maximize the value of big data for improving stroke outcomes. Figure 1 provides an overview of studies on stroke outcome using the *5Vs* framework. The *volume*, in terms of the sample analyzed, ranged from 1,073 to 167,640 participants. While a *variety* of stroke types (ischemic, hemorrhagic, undetermined stroke and TIA) and outcomes (multiple outcomes reported in one third of the studies) were explored, all but one of the studies were from high-income countries. The median *velocity* of data generation was 2,726 participants per year, including 9 studies with data on > 10,000 participants per year. However, *velocity* of data, in terms of median time between the end of data collection and generation of findings, was 6 years (interquartile range 4–7, maximum 17). *Veracity* of studies was examined, in terms of missing variables and biases, such as selection, misclassification, and recall bias. *Variability* was apparent based on the number of data sources explored (median 3 sources, interquartile range 1–11; Fig. 1).

## Outcomes After Stroke

### Mortality

Data on mortality after stroke were reported in 28 studies (*n* = 1,367 to 86,189 individuals) from 14 countries (Australia, Belgium, Canada, Denmark, France, Germany, Hong Kong, Italy, Korea, Netherlands, Norway, Sweden, UK, and USA) [8•, 9, 10, 11••, 12–20, 21•, 22••, 23•, 24••, 25–28, 29••, 30••, 31, 32••]. In these studies, mortality was ascertained using hospital administrative data [26, 27, 30••, 33], death registers [8•, 9, 10, 11••, 13–18, 20, 21•, 22••, 23•, 24••, 25, 28, 29••, 31, 32••, 34••, 35••, 36], or a combination of linkage with death registers and prospective follow-up assessments [12]. Cumulative crude rates of mortality post-stroke/TIA ranged from 7 to 18% within 30 days [9, 11••, 16, 20, 26] and 14 to 28% within 1 year [8••, 10••, 11••, 13, 20, 36]. In studies with longer-term data, 82% of patients with ischemic stroke died within 15 years [17]. Causes of death, mostly categorized as vascular or non-vascular, were reported in eight studies [11••, 17 , 24, 25, 27, 31, 32••].

Big data have also been explored to elucidate determinants of mortality, including patient- and system-level or healthcare factors. Patient sociodemographic and clinical factors are important considerations when examining post-stroke mortality. Although evidence on whether mortality rates after stroke/TIA differ by sex is equivocal [15, 36], other sociodemographic factors have been shown to be associated with greater risk of mortality post-stroke. These include low income [16, 26], poor educational attainment [16], and being unmarried and without children [28]. In one study undertaken in the USA, rates of mortality up to 10 years after intracerebral hemorrhage (ICH) were greater for White vs. non-White survivors [27]. Interestingly, in Canada, immigrants aged < 75 years had an 18% reduced risk of mortality over a median 5-year period than long-term residents [17]. However, immigrants from South Asia had increased mortality rates post-stroke than those who immigrated from other regions [17]. No difference was found in age-adjusted mortality rates among people with diabetes at 7, 30, and 365 days following stroke between First Nations and other residents in Canada [31].

The collection of standardized big data across multiple regions and jurisdictions allows investigation of geographical differences in mortality post-stroke. The effect of rurality on mortality was investigated in a Canadian study (*n* = 75,823) in which rural residents had a 7% greater risk of mortality during up to 5-year period of follow-up than their metropolitan counterparts [34••]. By contrast, no such difference was observed in Australia [23•]. In a novel finding, exposure to a low-dose particulate matter (PM_2.5_ < 12 µg/m^3^) over one year in the USA increased the risk of cardiovascular mortality by twofold during an average 6-year period after stroke [32••].

Similar to earlier reviews [4], stroke severity was identified as an important clinical predictor of mortality post-stroke [9, 12, 15]. Patients with less severe ICH, i.e., able to walk independently on admission, had about fourfold reduced risk of 180-day mortality than those with more severe ICH [21•]. Comorbidities associated with a greater risk of mortality post-stroke/TIA included history of atrial fibrillation/flutter [8•, 14, 27], cancer [8•, 21•], chronic kidney disease [14], diabetes [8•], dementia [14], depression [14], heart failure [8•, 14], hypertension [21•], myocardial infarction [8•], respiratory disease [14], or schizophrenia [11••, 20]. Current smoking was also reported to be associated with mortality post-stroke [21•]. Surprisingly, being overweight or obese was associated with a reduced risk of 1-year mortality after stroke [8•], the mechanisms of which remain unclear.

Using big data, authors have also identified COVID-19 infection as an emerging predictor of mortality post-stroke. In a nationwide study of German patients with stroke/TIA admitted across 1,463 hospitals, compared to the pre-pandemic period, inhospital mortality increased by 0.5% for ischemic stroke and by 5% for hemorrhagic stroke during the pandemic [37]. In another German study of 30,864 patients with acute ischemic stroke, rates of inhospital mortality were nearly threefold greater among those with concurrent COVID-19 infection than those without [30••]. Similarly, in a study of 41,588 patients hospitalized with stroke in France, patients with concurrent COVID-19 infection had an 85% increased rate of 3-month mortality [29••]. This finding was more pronounced in patients with ischemic stroke, with a twofold greater rate of 3-month mortality observed among those with concurrent COVID-19 than those without [29••].

A number of healthcare factors have been associated with improved survival after stroke/TIA. Receiving neuro-intervention following admission for stroke was associated with a 12% reduced risk of 3-month mortality [12], whereas early (vs. delayed) direct enteral feeding tube placement after acute stroke improved 30-day survival [25]. Using data from twelve hospitals in the USA, provision of one to two occupational or physical therapy sessions during admission for acute stroke was associated with a 30% reduced risk of 30-day mortality or readmission [26]. This effect was more pronounced among patients discharged to inpatient rehabilitation [26], a factor that has also been shown to be associated with improved 90-, 180-, and 365-day survival in Australia [13]. In contrast, receiving intravenous thrombolysis was not found to be associated with survival among 8,898 patients with ischemic stroke [18].

### Readmissions

Hospital readmission is an important outcome-based indicator of quality of care for conditions, such as stroke [38]. Readmissions were assessed in 10 studies (*n* = 1,998–116,073 individuals) from Australia [13, 33, 39, 40, 41••], the USA [26, 32••, 33, 42, 43], and Europe [14, 33]. Unadjusted cumulative rates of readmission following stroke ranged from 4.5% within 7 days [26], 7–13% (all stroke types) [26, 33, 42] or 16% (ischemic stroke only) [14] within 30 days, 25% within 90 days [40], and 53% within 1 year [13]. One in ten patients experience recurrence of ICH within 5 years of stroke; an estimate that was slightly greater for patients without atrial fibrillation [14]. The most common reasons for readmission include recurrence of cerebrovascular disease, cardiovascular disease, chest pain, abnormal findings, cancer, and gastrointestinal diseases [39, 40, 43]. Interestingly, more than 50% of patients were readmitted for reasons different from the initial hospitalization [33].

A number of patient-level factors were reported to be associated with a greater risk of readmission in the period up to 90 days after discharge for stroke. These included age [14], sex [42], place of residence or living situation [13, 14, 39], type of stroke [42], severity of stroke [14], functional independence or ability [39, 42], and poorer comorbidity profile [42]. Female sex, having a stroke of non-ischemic origin, greater Charlson comorbidity index scores, having an admission within 90 days before stroke, and having a poor functional ability on admission for stroke were associated with a greater risk of readmission [39, 42]. In a study of 9,255 Danish patients with ICH, greater age, not living at home, and having a more severe hemorrhage were associated with an increased risk of recurrent ICH within 5 years [14]. By contrast, history of diabetes, history of non-traumatic intracranial bleeding, and living alone (vs. cohabitating) were associated with a reduced recurrence of ICH [14].

Discharge destination remains an important factor influencing readmission. Among 8,555 Australian patients with acute stroke, those discharged to inpatient rehabilitation facilities from acute care were less likely to be readmitted within 1 year than patients discharged directly home, but were more likely to be readmitted than patients discharged to residential aged care [13]. By contrast, more frequent visits to a physical or occupational therapist were associated with a reduced risk of readmission or mortality within 30 days of discharge for stroke [26], with these effects being more pronounced in people discharged to post-acute care facility (vs. home) and in those having problems (vs. no problems) with mobility [26].

### Medication Use or Adherence

Secondary prevention medications are strongly recommended for survivors of stroke to reduce their risk of subsequent vascular events [44, 45]. Despite these recommendations, disparities in the use of medications were highlighted in several big data studies [46••, 47, 48]. In one such study undertaken among 9,817 Australian patients with first-ever stroke/TIA, about one in five patients never filled a prescription for an antihypertensive, antithrombotic, or lipid-lowering medication within 1 year of hospital discharge [46••]. Of greater concern, up to one-third of patients who initially filled a prescription subsequently discontinued therapy for ≥ 90 days (21% discontinued antihypertensive medications, 34% antithrombotic medications, and 29% lipid-lowering medications) [46••]. Factors associated with continuation of antihypertensive medications included having a prescription at discharge from hospital, having quarterly contact with a primary care physician, and being prescribed medications by a specialist [46••]. In other studies, being female [47] and having greater psychological distress [48] were associated with suboptimal medication adherence after stroke. The importance of medication adherence on survival was highlighted in another study of 8,363 Australian survivors of stroke/TIA [22]. In that study, among those with adherence levels ≥ 60% during the first year after stroke, a 10% increase in medication adherence was associated with a 13–15% linear reduction in mortality during the subsequent 2 years [22••].

### Functional Measures

Recovery and return to usual daily activities rely heavily on improvements in functional outcomes after stroke. Functional outcomes were reported in five studies, with sample sizes ranging from 1,367 to 35,913 adults [10••, 25, 36, 41••, 49]. In these studies, functional outcomes were captured in registry, hospital, or pharmaceutical claims data, using a variety of scales, such as the modified Rankin Scale (mRS) [10••, 25, 41••, 50], the functional independence measure [49], and ability to undertake activities of daily living (i.e., toileting or dressing) [10••, 36]. Among Australian patients admitted to an inpatient rehabilitation facility following acute stroke/TIA, those with greater relative functional gain (defined by a change in functional independence measure) during rehabilitation were ≥ 10 times more likely to be independent (mRS 0–2) at 90–180 days after stroke/TIA [41••]. Similarly, in a Swedish cohort, having ≥ 2 prescriptions (for either anticoagulant, antihypertensive, antidepressant, or diabetes medications) in the year before ischemic stroke was associated with 50–100% greater odds of being independent (estimated mRS 0–2 or ability to toilet or dress) [10••].

Researchers have also explored the potential utility of home-time as a surrogate for functional outcomes [50], as discussed in the section on validation studies below.

### Quality of Life

Health-related quality of life (HRQoL) after stroke is an important outcome measure for understanding the health status and any impairments experienced by people living with stroke/TIA in the community. However, the utility of big data to understand HRQoL after a stroke/TIA is yet to be fully explored globally. Reports of big data on HRQoL outcomes post-stroke/TIA came from five studies that were based on data from the Australian Stroke Clinical Registry (*n* = 4,239 to 28,115 survivors), collected during follow-up surveys administered between 90 and 180 days after acute stroke/TIA [13, 23•, 39, 41••, 51•]. In these studies, HRQoL was self-reported via the EuroQoL five-dimensional 3-level questionnaire [52]. Among registrants with a first-ever stroke, 60% reported problems with mobility and usual activities, 50% with anxiety/depression and pain, and 30% with self-care [39].

Researchers have also explored whether HRQoL at 90–180 days post-stroke/TIA varied by demographic characteristics [23•, 51•]. No sex difference was observed in the overall HRQoL, assessed using a visual analogue scale ranging from 0 to 100 (worst to best imaginable health) [51•]. However, females were 8% to 16% more likely to report problems related to usual activities, anxiety/depression, or pain/discomfort than males [51]. Compared to patients treated in rural hospitals, those treated in metropolitan hospitals more often reported extreme problems with mobility (7% *vs.* 5%) and self-care (12% vs 9%) [23•].

Discharge destination and functional gain during rehabilitation care were associated with HRQoL at 90–180 days post-stroke/TIA [13, 41••]. Compared to survivors who were discharged home, the overall HRQoL was worse by 6.5 points for those discharged to rehabilitation [13]. However, survivors discharged to rehabilitation had better overall HRQoL than those discharged to residential aged care (+ 43.4 points) [13]. In another study, there was a 22-point gain in HRQoL among those with greater relative functional gain (defined by a change in functional independence measure) [41••].

### Healthcare Costs

The economic burden of stroke is considerable, with annual direct and indirect costs estimated to exceed €60 billion in Europe [53] and AU$32 billion in Australia [54]. With limited healthcare resources, large, routinely collected data on the use of healthcare services are instrumental for eliciting potential opportunities for cost containment. Costs after stroke, or associated factors, were reported in four studies conducted in Canada, China, Sweden, and the USA (*n* = 1,073 to 29,673) [55–58]. Among patients with stroke and systemic embolism with non-valvular atrial fibrillation taking oral anticoagulants, healthcare costs were greater among those with major bleeding (US$81,414; mean 343 days after stroke) than those without (US$32,607; mean 327 days after stroke) [58]. In a cohort of 1,073 Swedish patients with acute ischemic stroke, the cost of inpatient rehabilitation was significantly lower among those with dementia (US$7,752) than those without dementia (US$12,239) [57]. Among 3,673 people with stroke in Canada, overall costs of healthcare utilization were driven by the level of comorbidity and not the index stroke event [55]. Costs were largely due to post-acute care services for those with low levels of comorbidity, and acute care services for those with high levels of comorbidity [55].

## Big Data Analytics for Stroke Outcomes

Big data are often complex. Therefore, advanced analytical methods, such as machine learning, are increasingly being used to complement standard statistical methods [3], in order to navigate the nuances and complexities of big data. Moreover, big data often lack some clinically important predictors of stroke outcome (e.g., level of frailty, stroke severity) that are difficult, expensive, or not feasible to collect. As a result, sophisticated analytic methods are being used to validate surrogate variables for these measures. In this section, we review advanced analytical methods reported in recent studies of stroke outcomes or their determinants.

### Machine Learning

Machine learning methods optimize technological advances in computing power and statistical tools for analyzing big data, and are driving advances in person-centered care [59]. Advanced machine learning algorithms, capable of handling multi-collinearity, non-linearity, and higher order interactions among variables, are being used to identify novel predictors of patient outcomes beyond those identified via conventional regression analyses [60].

Various machine learning algorithms have been developed for predicting mortality and functional outcomes post-stroke, with varying degrees of accuracy [61]. The most popular machine learning methods being used to improve the prediction of stroke outcomes are random forests, support vector machines, decision trees, and deep neural networks [59]. In one study, random forests performed best for identifying predictors of 30-day mortality after stroke [62••]. Models involving natural language processing of unstructured free-text magnetic resonance imaging reports have been successfully used to predict 90-day functional outcomes with great accuracy [63]. Machine learning methods have also been used to predict other stroke outcomes, including 30-day readmissions [60, 64], home-time [50], motor recovery [65], and complications of stroke (e.g., pneumonia [66], and dysphagia [67]) and for developing a proxy measure of stroke severity [68]. Also, machine learning methods have been used to maximize the utility of longitudinal measures collected after acute stroke for improving the prediction of outcomes [65, 66, 68, 69]. These include the use of 30-day mRS scores to predict 90-day mRS scores [68], longitudinal measures of medication use or pathological measures to predict hospital-acquired pneumonia [66], or combining sequential data on upper extremity capacity recovery to predict post-stroke motor recovery [65]. Despite these advances, there remains a need to address issues related to missingness of data, internal validation, reporting of model development, configuration, features, discrimination and calibration, and sample size limitations [59, 61].

### Validation of Surrogate Measures Using Big Data

Big data studies often lack information on important clinical or biological measures, such as patient frailty [35••] or stroke severity [19, 70, 71], that are pertinent to the research of stroke outcomes. To overcome this limitation, authors have developed surrogate indicators for these measures using other routinely available prognostic variables. Examples of these newly developed surrogate measures are discussed in further detail below. These recent advances allow for more robust assessment of stroke outcomes and promote better standardization of methods in research using big data.

Stroke severity is one of the most important factors for predicting outcomes post-stroke [9, 12, 15], but information on this measure is often unavailable in big datasets. In the Australian Stroke Clinical Registry, information is routinely collected on a patient’s ability to walk independently on admission to provide a simple, validated marker of stroke severity that is predictive of 30-day mortality and mRS scores [39, 72]. In studies where information on stroke severity cannot be obtained through data linkage with a stroke registry, alternative surrogate measures are commonly used to infer stroke severity, such as the Glasgow Coma Scale [9, 14], mode of arrival to hospital [9], or length of stay in hospital [28]. Moreover, researchers have recently developed a new measure of stroke severity that is predictive of 30-day mortality using a combination of variables routinely collected in big data, including age, sex, arrival by ambulance, transfer to a stroke hospital, use of mechanical ventilation, and triage scores [19].

Other new surrogate measures of stroke outcome developed for use in big data include the *hospital frailty risk score*, the *FRAC-Stroke score*, and the *home-time* measure. The hospital frailty risk score is derived using International Statistical Classification of Diseases and other Related Health Problems 10th revision (ICD-10) codes for 109 conditions associated with frailty, including cognitive impairment, delirium, consciousness, falls, and urinary incontinence [35••]. This measure has been shown to be valid and reliable for predicting multiple poor outcomes after stroke, including 30-day mortality, 90-day readmissions, and HRQoL between 90 and 180 days [35••]. The FRAC-Stroke score differs from the hospital frailty risk score as it can be used to identify patients at elevated risk of low-trauma fracture after stroke who could benefit from pharmacotherapy or bone densitometry screening [73••].

Home-time is defined as the number of days after stroke admission that a patient is alive and not hospitalized [74–76], and may be considered a more pragmatic indicator of global disability than examining individual components of readmissions and mortality. During the first 90 days post-stroke, an average 55 to 60 days are spent at home for people with ischemic stroke [74, 77], 49 days after subarachnoid hemorrhage [74], and 0 days after ICH [74, 77]. Greater stroke severity [74, 76], older age [18, 77], and reduced functional recovery [74–77] are all associated with reduced 90-day home-time. Receiving endovascular treatment may increase 90-day home-time by 10 days among those with ischemic stroke [18]. However, despite the good external validity of the home-time measure, heterogeneity in health service practices across jurisdictions may lead to variations in its estimation and limit its generalisability.

Use of big data for pharmaco-epidemiology can also present additional methodological challenges for researchers [78••]. For example, the calculation of medication adherence in big data requires information on the intended duration of each prescription (known as the prescribed daily dose). However, this information is often not collected in prescription claims data, necessitating imputation of information on dose from other sources. In a recent exploratory study, imputing one dose per day for antihypertensive medicines (excluding beta-blockers) yielded similar estimates for 1-year adherence compared to a more complicated method involving the 75th percentile of refill time [78••]. Continued standardization and transparent reporting of pharmaco-epidemiological methods is needed to ensure findings from big data studies are robust and generalizable.

## Future Directions

Despite increased capacity to generate big data on stroke outcomes, there remain considerable delays between data generation and publication, thereby limiting the value of these data for driving real-time improvements in patient outcomes. Although machine learning methods have helped maximize the efficiency of big data for improving the prediction of stroke outcomes, greater rigor is needed in the reporting of these methods to allow their evaluation and external validation prior to more widespread adoption [59, 61]. Lack of studies from low- to middle-income countries highlights opportunities to develop the capacity to generate and harness big data for improving stroke outcomes in these settings. Also, there is a need to explore big data to unearth insights on stroke outcomes in rare patient groups and less-studied populations.

## Conclusion

Ultimately, there have been many recent advances in the use of big data to explore outcomes after stroke, including mortality, readmission, medication use or adherence, functional outcomes, quality of life, and health costs. Based on the *5Vs* framework, further work is needed to improve the *variety*, *velocity*, and *veracity* of big data–enabled research. Big data are driving rapid innovations in research of stroke outcomes and generating novel evidence to bridge knowledge and practice gaps.

## Supplementary Information

Below is the link to the electronic supplementary material.

Supplementary file1 (DOCX 44.5 KB)
