# Supplementary material for: The Allure of Big Data to Improve Stroke Outcomes: Review of Current Literature
Source: Curr Neurol Neurosci Rep. 2022 Mar 11;22(3):151–60. doi: 10.1007/s11910-022-01180-z (PMC8913242; doi:10.1007/s11910-022-01180-z)
Supplement: Supplementary file 1 — Supplementary file1 (DOCX 44.5 KB) [file 11910_2022_1180_MOESM1_ESM.docx]

**Supplementary materials**

| **Table I. Characteristics of the studies reporting stroke outcomes** | | | | | | | | | | | | | |
| --- | --- | --- | --- | --- | --- | --- | --- | --- | --- | --- | --- | --- | --- |
| **S/N** |   **Author (Ref #) ^a^** | **Country** | **Study period** | **Follow-up duration** | **Stroke type** | **Sample analyzed (n)** | **Outcomes** | **No. of datasets** | **Type of datasets** | | | | |
|  |  |  |  |  |  |  |  |  | **Registry** | **Hospital** | **Pharma** | **Mortality** | **Other** |
| 1 | Andrew (39) | Australia | 2009-2013 | 90-180 days | AIS, ICH, TIA | 7,183 | Readmission, HRQoL | 3 | x | x |  | x |  |
| 2 | Barrett (10) | Australia | 2012-2017 | 3, 6, 9, 12 months | AIS, TIA | 4,209 | Medication use | 3 |  | x | x | x |  |
| 3 | **··**Dalli (46) | Australia | 2010-2014 | 1 years | All, TIA | 17,980 | Medication adherence | 3 | x |  | x |  | x |
| 4 | **··**Dalli (22) | Australia | 2010-2014 | 3 years | All, TIA | 8,363 | Medication adherence,  Mortality | 3 | x |  | x | x |  |
| 5 | **·**Dwyer (23) | Australia | 2010-2015 | 90-180 days | AIS, ICH | 28,115 | Mortality, HRQoL | 3 | x |  |  | x | x |
| 6 | Gattellari (9) | Australia | 2011-2014 | 1 month | AIS | 17,700 | Mortality | 3 |  | x |  | x | x |
| 7 | Kilkenny (40) | Australia | 2009-2013 | 90 days | AIS, ICH, UND, TIA | 13,594 | Readmission | 3 | x | x |  | x |  |
| 8 | Lynch (13) | Australia | 2010-2013 | 3, 6, 12 months | AIS, ICH, UND | 8,555 | Mortality, Readmission, HRQoL | 3 | x | x |  | x | x |
| 9 | **··**Mosalski (41) | Australia | 2014-2017 | 90-180 days | AIS, ICH, UND | 4,239 | mRS, HRQoL, Readmissions | 3 | x | x |  |  | x |
| 10 | **·**Phan (51) | Australia | 2010-2014 | 90-180 days | AIS, ICH, UND | 6,852 | HRQoL | 3 | x | x |  |  | x |
| 12 | Phan (15) | Australia | 2010-2014 | 7 days, 1 & 12 months | AIS, ICH, UND | 14,118 | Mortality | 3 | x | x |  | x |  |
| 12 | Welsh (48) | Australia | 2006-2009 | 3 months | AIS, TIA | 1,323 | Medication adherence | 4 |  | x | x | x |  |
| 13 | **·**Zhang (21) | Australia | 2009-2013 | 90-180 days | AIS, ICH, TIA, UND | 2,098 | Mortality | 3 | x | x |  | x |  |
| 14 | **··**Burneo (24) | Canada | 2003-2009 | 2 years | All | 19,138 | Epilepsy | 11 | x | x | x | x | x |
| 15 | Griffith (55) | Canada | 2008 | 5 years | All | 376,367 | Costs | 5 |  | x | x |  | x |
| 16 | Joundi (25) | Canada | 2003-2013 | 30 days | AIS, ICH | 1,367 | Mortality, mRS | 2 | x |  |  | x | x |
| 17 | **··**Kapral (11) | Canada | 2002-2013 | 7, 30, 365 days | AIS | 52,473 | Mortality | 3 | x | x |  |  | x |
| 18 | **··**Kapral (34) | Canada | 2008-2012 | 5 years | All | 6,282,855 | Mortality | 6 |  | x | x |  | x |
| 19 | Kapral (31) | Canada | 2011-2016 | 7, 30, 365 days | All, TIA | 28,874 | Mortality | 2 |  | x |  |  | x |
| 20 | Vyas (17) | Canada | 2002-2013 | 15 years + | AIS | 28,148 | Mortality | 3 | x | x |  | x |  |
| 21 | Yu (18) | Canada | 2002-2013 | 1 year | AIS | 8,898 | Mortality, Home-time | 2 | x | x |  |  | x |
| 22 | Yu (36) | Canada | 2011 -2016 | 7 years | AIS, HEM | 4,831 | Functional Outcomes ^b^, Mortality | 3 |  | x |  |  | x |
| 23 | Qiao (56) | China | 2009 | N/A | All | 3,590 | Costs | 1 |  |  |  |  | x |
| 24 | Overvad (14) | Denmark | 2003-2016 | 5 years | ICH | 9,255 | Mortality, Readmission | 4 | x | x | x | x |  |
| 25 | **··**Gabet (29) | France | 2020 | 3 months | All | 56,195 | Mortality | 1 |  |  | x |  |  |
| 26 | **··**Ritcher(30) | Germany | 2020 | N/A | AIS | 68,913 | Mortality | 1 |  |  | x |  |  |
| 27 | Richter (37) | Germany | 2021 | 2 months | AIS, ICH, TIA | 47,983 | Mortality | 1 |  | x |  |  |  |
| 28 | Yung (20) | Hong Kong | 2006-2016 | 30 days; 1 year | AIS | 8,987 | Mortality | 1 |  |  |  |  | x |
| 29 | Kim (12) | Korea | 2007-2014 | 3 months | AIS | 1,405 | Mortality | 2 | x |  |  |  | x |
| 30 | Skyrud (16) | Norway | 2005-2009 | 30 days | All | 45,448 | Mortality | 3 | x | x |  |  |  |
| 31 | Hoang (57) | Sweden | 2010-2014 | N/A | All | 7,383 | Costs | 2 | x |  |  |  |  |
| 32 | **··**Ingrid(10) | Sweden | 2009-2011 | 1, 2 years | AIS | 35,913 | Mortality, mRS, ADL | 3 | x |  | x | x | x |
| 33 | Meyer (28) | Sweden | 1998-2002 | 12 years | AIS | 70,149 | Mortality | 3 |  | x |  |  |  |
| 34 | Brown (49) | USA | 2005-2007 | 3 months | All | 16,346 | FIM | 1 |  |  |  |  | x |
| 35 | **·**Chaudhary (8) | USA | 2003-2019 | 1 years | AIS | 6,015 | Mortality | 4 | x |  |  | x | x |
| 36 | Daras (42) | USA | 2013-2014 | 30 days | All | 116,073 | Readmission | 2 |  | x |  |  | x |
| 37 | Freburger (26) | USA | 2016-2018 | 7, 30 days | All | 8,279 | Mortality, Readmission | 2 |  | x |  |  | x |
| 38 | Kuohn(27) | USA | 2005-2014 | 10 years | ICH | 72,432 | Mortality, Readmission | 1 |  | x |  |  |  |
| 39 | **··**Liao (32) | USA | 2007-2016 | 10 years + | All | 54,723 | Mortality, Readmission | 1 |  | x |  |  |  |
| 40 | Raja (43) | USA | 2013 | 1 month | AIS, HEM | 1998 | Readmission | 1 |  | x |  |  |  |
| 41 | Rozjabek (58) | USA | 2009-2016 | 1, 2, 3, 4 years | AIS | 1,340 | Costs | 1 |  |  |  |  | x |
| 42 | Brunner-LaRocca (33) | Multiple ^c^ | 2009-2014 | 1 month | All | 86,502 | Mortality, Readmission | 1 |  | x |  |  |  |
| TIA, transient ischemic attack; AIS, acute ischemic stroke; HEM, hemorrhagic stroke; ICH, intracerebral hemorrhage; SAH, subarachnoid hemorrhage; UND, undetermined stroke; HRQoL, health-related quality of life; mRS, modified Rankin scale; ADL, activities of daily living; FRAC-Stroke, fracture risk after ischemic stroke; FIM, functional independence measure; UK, United Kingdom; USA, United States of America; N/A, not available.  **·**Important; **··**Very important.  ^a^ First authors name and reference number mentioned; ^b^ Multiple functional outcomes were assessed; ^c^ Comprised participants from seven countries, including United States, United Kingdom, Australia, and Continental Europe (Belgium, Denmark, Italy and the Netherlands). | | | | | | | | | | | | | |
